# ^18^F-Fluoride PET/CT tumor burden quantification predicts survival in breast cancer

**DOI:** 10.18632/oncotarget.16418

**Published:** 2017-03-21

**Authors:** Ana E. Brito, Allan Santos, André Deeke Sasse, Cesar Cabello, Paulo Oliveira, Camila Mosci, Tiago Souza, Barbara Amorim, Mariana Lima, Celso D. Ramos, Elba Etchebehere

**Affiliations:** ^1^ Nuclear Medicine Division, Campinas State University (UNICAMP), SP, Brazil; ^2^ MND Campinas, Campinas, SP, Brazil; ^3^ Department of Internal Medicine, Campinas State University (UNICAMP), SP, Brazil; ^4^ Department of Gynecology and Obstetrics, Campinas State University (UNICAMP), SP, Brazil; ^5^ Department of Statistics, Campinas State University (UNICAMP), SP, Brazil

**Keywords:** fluoride-PET/CT, NaF PET/CT, breast cancer, skeletal tumor burden, bone metastases

## Abstract

**Purpose:**

In bone-metastatic breast cancer patients, there are no current imaging biomarkers to identify which patients have worst prognosis. The purpose of our study was to investigate if skeletal tumor burden determined by ^18^F-Fluoride PET/CT correlates with clinical outcomes and may help define prognosis throughout the course of the disease.

**Results:**

Bone metastases were present in 49 patients. On multivariable analysis, skeletal tumor burden was significantly and independently associated with overall survival (*p* < 0.0001) and progression free-survival (*p* < 0.0001). The simple presence of bone metastases was associated with time to bone event (*p* = 0.0448).

**Materials and Methods:**

We quantified the skeletal tumor burden on ^18^F-Fluoride PET/CT images of 107 female breast cancer patients (40 for primary staging and the remainder for restaging after therapy). Clinical parameters, primary tumor characteristics and skeletal tumor burden were correlated to overall survival, progression free-survival and time to bone event. The median follow-up time was 19.5 months.

**Conclusions:**

^18^F-Fluoride PET/CT skeletal tumor burden is a strong independent prognostic imaging biomarker in breast cancer patients.

## INTRODUCTION

Bone metastasis is a common cause of serious morbidity in patients with breast cancer. It is associated with various debilitating skeletal-related events, which include bone fractures, hypercalcemia, nerve compression, and severe pain. The diagnosis of bone metastasis influences the patient's prognosis, reducing overall survival (OS) [1]. The early detection of bone metastases in newly diagnosed breast cancer patients is important because it changes the ideal treatment strategies [2–4]. Recent guidelines recommend that stage IIIA breast cancer patients should undergo staging with either conventional bone scintigraphy or with ^18^F-Fluoride PET/CT [5]. While both ^18^F-fluoride (PET/CT) and ^99m^Tc-MDP (conventional bone scintigraphy) are bone-seeking tracers used to identify bone remodeling and detect areas of increased bone remodeling due to metastases [6], when comparing the two imaging modalities for staging and restaging breast cancer patients, clearly ^18^F-fluoride PET/CT is ideal due to greater sensitivity, specificity and accuracy [7]. Furthermore, ^18^F-Fluoride PET/CT has been shown to alter treatment plan in approximately 39% of breast cancer patients [8].

Beyond lesion detection and staging, it is feasible to quantify skeletal tumor burden using ^18^F-Fluoride PET/CT. Determination of skeletal tumor burden has been shown to have a prognostic role in prostate cancer patients treated with ^223^Ra [9].

Studies have shown that calculation of the primary tumor metabolism using parameters such as total lesion glycolysis (TLG) and metabolic tumor volume (MTV) on ^18^F-FDG PET/CT images predicts survival in breast cancer patients at initial staging [10, 11]. However, when breast cancer patients develop bone metastases, there are no means to foresee which patients will have a shorter survival time. Even though breast cancer bone metastases are ^18^F-FDG-avid, unfortunately, quantification of whole-body tumor burden with this tracer is not practical because the areas of normal biodistribution.

Only one recent study investigated the prognostic role of ^18^F-Fluoride PET/CT in breast cancer patients semi-quantitatively [12]. While the authors did not find a significant correlation, the parameters that they used did not evaluate the entire bone disease extent on ^18^F-Fluoride images. To that effect, there are no studies that calculated the entire skeletal tumor burden turnover on ^18^F-Fluoride PET/CT and correlated with prognosis in breast cancer patients.

The purpose of this study was to correlate skeletal tumor burden determined by ^18^F-Fluoride PET/CT with clinical outcomes in breast cancer patients.

## RESULTS

### Patient characteristics

A total of 107 female patients, mean age 59.6 ± 13.3 years and a mean of 4.1 ± 4.9 years from primary diagnosis (0.1 – 20.3 years) were studied (Table 1). The median follow-up time was 19.5 months (2 - 83 months). Among the 107 patients studied, twenty-three patients died and, among these, two died very early after performing the ^18^F-Fluoride PET/CT study (2 and 7 months afterwards). Histology consisted of 91 (85%) invasive ductal carcinomas, 12 (11.2%) invasive lobular carcinomas, 3 adenocarcinomas (2.8%) and 1 sarcoma (1%). According to the TNM staging system, 30 (28%) patients were stage I, 29 (27%) were stage II, 35 (33%) were stage III and 13 (12%) had stage IV disease.

**Table 1:** Clinical characteristics of patients

		*N* or median	% or range
**Age**		59.6	30–93
**Years of cancer**		4.0	0.1–20.3
**PR positive**		69	64%
**ER positive**		78	73%
**Histology type**	Ductal	91	85%
	Lobular	12	11.2%
	Adenocarcinoma	3	2.8%
	Others	1	< 1%*
**Her-2 expression**		14	13%
**TNM stage at diagnosis**	I	30	28%
	II	29	27.1%
	III	35	32.7%
	IV	13	12.1%
**Previous treatments**	chemotherapy	82	77%
	radiotherapy	53	50%
	surgery	57	53%
	hormone therapy	87	81%
	no treatment	2	1,9%

ER = estrogen receptor; PR = progesterone receptor; * = sarcoma.

The patients were submitted to ^18^F-Fluoride PET/CT for detection of bone metastases. Forty patients underwent ^18^F-Fluoride PET/CT for primary staging of breast cancer. The remainder underwent ^18^F-Fluoride PET/CT with suspicion of bone metastases prior to or after some modality of treatment. The treatment consisted of one or more of the following: chemotherapy (82 patients), radiotherapy (53 patients), surgery (57 patients) and hormone therapy (87 patients).

Among the 107 patients enrolled, 49 patients (45.8%) were diagnosed with bone metastases. Analyzing only the population that performed the ^18^F-Fluoride PET/CT for staging, 32.5% (13 patients) were positive for bone metastasis.

The analysis of the tumor burden of these 49 patients was undertaken and compared to the 58 patients without bone, visceral or nodal metastases. Nineteen patients (17.7%) had visceral metastases (15 patients with lung metastases and 4 patients with liver metastases) at the time of the ^18^F-Fluoride PET/CT examination. All patients with hepatic lesions and 12 patients with lung lesions had also bone metastases. Thus, 16 patients (15%) had bone and visceral metastases. Although all patients had undergone CT scans of the chest, abdomen and pelvis for detection of visceral metastases, 20 patients (18.7%) also underwent an ^18^F-FDG PET/CT study within 3 months of the ^18^F-Fluoride PET/CT. In these cases, the ^18^F-FDG PET/CT exams were also considered when evaluating for visceral metastases.

### Skeletal tumor burden (TLF_10_) analysis of the 49 patients with bone metastases

^18^F-Fluoride PET/CT images detected bone metastasis in 49 (45.8%) patients. The *h*SUV of the bone metastases for all patients (mean ± SD) was 46.7 ± 23.37 (range 12.6 - 96.5) and the Mean_10_ for all patients (mean ± SD) was 14.8 ± 5.2 (range 9.4 - 43.2). The mean FTV_10_ was 204.1 ml (range 0.5–1578 ml) and the mean TLF_10_ was 3395.3 (range 9.0–39410). TLF_10_ and FTV_10_ values were highly correlated (*p* = 0.95; *P* < 0.0001) and therefore only TLF_10_ was used for further analyses.

### TLF_10_ and OS

At the end of the follow-up period, 84 patients were alive (30 with bone metastasis). The median overall survival was 15.2 months for patients with bone metastasis and 23.4 months for patients without bone metastasis.

TLF_10_ was significantly associated with OS on univariable analyses (*p* < 0.0001; HR = 1.136; 95% CI = 1.066–1.210). The presence of bone or visceral metastasis, *h*SUV, negative progesterone receptor (PR) *status* and ECOG *status* were also correlated with survival in the univariate analysis. Other parameters such as initial tumor characteristics (HER2 *status*, ER *status*, Ki-67 index), the current patient’s age, the time of disease, ECOG *status*, current pain score, and treatments (surgery, chemotherapy and radiotherapy) during the course of disease did not correlate with OS.

On multivariable analyses TLF_10_ (*p* < 0.0001; HR = 1.136; 95% CI = 1.062–1.216) and negative PR *status* of the primary tumor (*p* = 0.0025; HR = 4.648; 95% CI = 1.575–13.718) were the only two parameters significantly associated with OS (Table 2).

**Table 2:** Correlation of clinical, laboratory and imaging variables to overall survival

Variables	HR	95% CI		*p*-value
	Univariable analyses			
Age	0.998			0.9131
Time of disease	1.011	0.929	1.099	0.8046
Primary stage (III/IV vs I/II)	1.117	0.423	2.953	0.8230
HER2	1.019	0.388	2.674	0.9697
ER *status*	1.763	0.578	5.379	0.3192
PR *status*	4.078	1.513	10.991	**0.0055**
Ki-67	1.020	0.999	1.042	0.095
Radiotherapy (y/n)	1.140	0.445	2.917	0.7853
Hormonal therapy (y/n)	1.783	0.657	4.831	0.2561
Surgery (y/n)	2.067	0.762	5.604	0.1539
ECOG *status*	4.101	1.517	11.083	**0.0054**
Pain score	2.537	0.991	6.496	0.0524
Visceral metastases	5.181	2.488	10.799	**< 0.0001**
Bone metastases	6.461	2.190	19.062	**0.0007**
*h*SUV	1.022	1.005	1.041	**0.0139**
Mean_10_	0.927	0.882	1.084	0.6647
TLF_10_	1.136	1.066	1.210	**< 0.0001**
	**Multivariable analysis**			
TLF_10_	1.136	1.062	1.216	**< 0.001**
PR *status*	4.648	1.575	13.718	**0.0025**

y/n = yes versus no; OS = Overall survival; HR = Hazard ratio ; CI = Confidence interval ; PR = progesterone receptor; ER = estrogen receptor.

The patient group that underwent ^18^F-Fluoride PET/CT examination for staging had a median TLF_10_ of 4376.7 (SD = 1078.2; Minimum = 9.0; Maximum = 39,409.8). Likewise, the patient group that underwent ^18^F-Fluoride PET/CT examination for restaging had a median TLF_10_ = 3040.9 (SD = 4572.6; Minimum = 10.2; Maximum = 4,950.1). When comparing the staging and re-staging groups in terms of OS, PFS and TLF10 values there were no significant differences (*p* = 0.4894, p = 0.1593, *p* = 0.3591).

**Figure 1:**
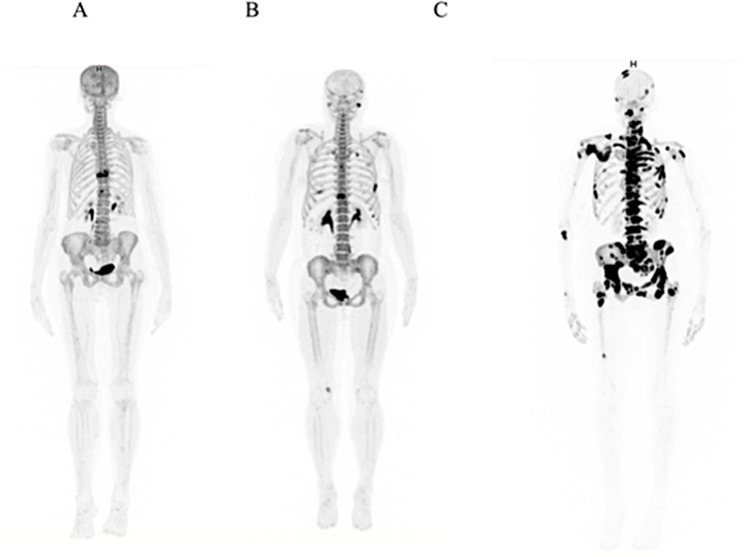
^18^F-Fluoride PET/CT images of three women demonstrating differences in skeletal tumor burden extent and the outcome (**A**) Image of a 70 yr-old patient with ductal breast cancer for 2.6 yrs demonstrating a metastasis in the 9^th^ thoracic vertebrae on the ^18^F-Fluoride PET/CT. The skeletal tumor burden metrics was low (TLF_10_ = 641) and the patient remained 21 months with stable disease and event-free. (**B**) Image of a 43 yr-old woman with ductal breast cancer, diagnosed 3 months prior to ^18^F-Fluoride PET/CT images, demonstrating bone metastases in the spine, ribs and left temporal bone. Her skeletal tumor burden was intermediate (TLF_10_ = 1039.7) and although the patient indeed progressed, she was still alive after 17 months. (**C**) Image of a 63 yr-old woman with ductal breast cancer, diagnosed 4 months ago, and multiple metastasis. The skeletal tumor burden was extremely high (TLF_10_ = 39409). The patient progressed in 1.5 months and died in 2 months.

**Figure 2:**
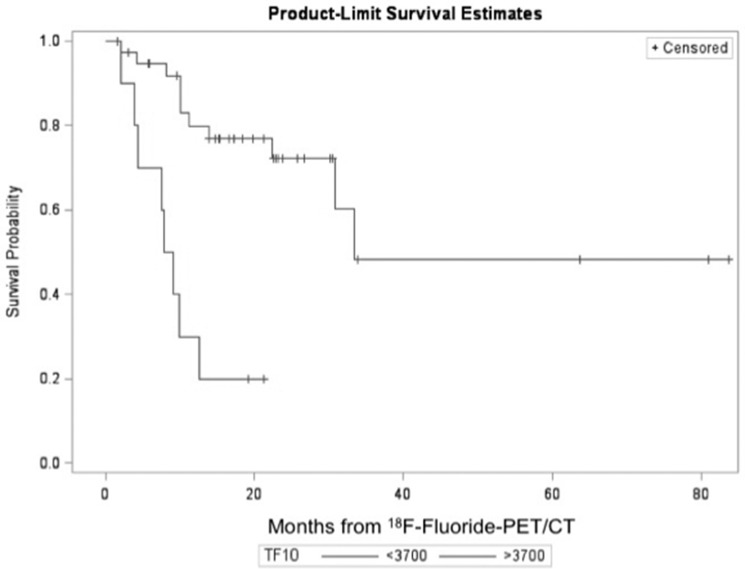
Overall survival according to TLF_10_ on ^18^F-Fluoride PET/CT For TLF_10_ < 3700 the mean OS = 26.94 months (SD = 1.87) and median OS = 33.43 months. For TLF_10_ > 3700 the mean OS = 8.26 months (SD = 1.25) and median OS = 8.48 months.

Higher TLF_10_ values (meaning more metastases) were associated with worst survival (Figure 1). A TLF_10_ cutoff of 3,700 separated two groups in terms of survival. Patients with TLF_10_ > 3,700 had a significantly higher risk of death (median OS = 8.5 months) while patients with TLF_10_ < 3,700 had a median OS of 33.4 months (*p* = 0.0002; HR = 6.569; 95% CI = 2.419–17.835) (Figure 2).

### TLF_10_ and PFS

At the end of follow-up, 32 patients (30%) progressed (eight had bone progression, four had nodal progression, 13 had visceral progression and seven had an increase in ECOG score by 2 points). Visceral metastases were located in the lungs and liver. Among these patients, 27 had bone metastasis prior to progression, one patient had a liver metastasis and the remaining four patients were disease-free. The most common site of progression of the 27 patients with known bone metastasis was visceral disease. Visceral (lung and liver) metastases occurred in 13 patients.

The median PFS for patients with *vs* without bone metastases was 4.7 *vs* 12.2 months, respectively. Analyzing only the 49 patients with bone metastases at the baseline ^18^F-Fluoride PET/CT scan, the mean TLF_10_ was 2.5 times greater for patients that progressed when compared to those that did not progress (TLF_10_ = 4,670 *vs* 1,831).

**Figure 3:**
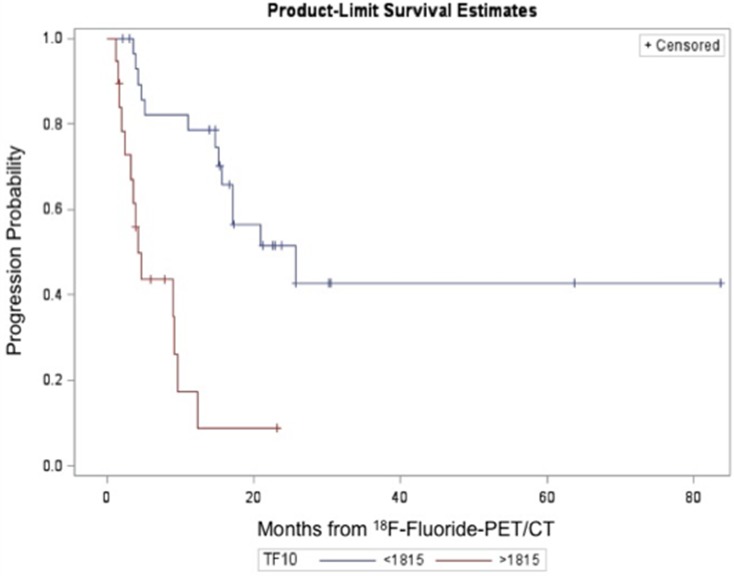
Progression probability according to TLF_10_ on ^18^F-Fluoride PET/CT For TLF_10_ < 1815 the mean PFS = 19.01 months (SD = 1.66) and median PFS = 25.80 months. For TLF_10_ > 1815 the mean PFS = 6.16 months (SD = 0.99) and median PFS = 4.13 months.

TLF_10_ was associated with PFS on univariable analyses (*p* < 0.0001; HR = 1.131; 95% CI = 1.068–1.198). The presence of bone metastases, visceral metastasis, negative progesterone receptor (PR), age and ECOG *status* also significantly correlated with PFS in the univariable analyses. All other parameters (HER2 *status*, ER *status*, Ki-67 index, time of disease, pain score, and treatments during the course of disease) did not correlate with PFS. On multivariable analyses however, TLF_10_ (*p* < 0.0001; OR = 1.120; 95% CI = 1.058–1.187) and a negative PR primary tumor *status* (*p* = 0.0413; HR = 2.266; 95% CI = 1.015–5.061) were again the only parameters associated with PFS (Table 3). Higher TLF_10_ values (meaning more metastases) were associated with higher risk of progressing (Figure 3). A TLF_10_ cutoff of 1,815 separated two groups in terms of progression (25.8 vs 4.13 months) (*p* = < 0.0001; HR = 5.384; 95% CI = 2.339–12.395).

**Table 3:** Correlation of clinical, laboratory and imaging variables to progression-free survival

Variables	HR	95% CI		*p*-value
	Univariable analyses			
Age	0.970	0.941	0.999	**0.0424**
Time of disease	0.987	0.923	1.058	0.7110
Primary stage (III/IV vs I/II)	1.946	0.836	4.528	0.1225
HER2	1.657	0.792	3.511	0.1874
ER *status*	1.301	0.494	3.423	0.5939
PR *status*	2.808	1.323	5.957	**0.0071**
Ki-67	1.015	0.998	1.032	0.0868
Radiotherapy (y/n)	1.485	0.688	3.208	0.3138
Hormonal therapy (y/n)	1.002	0.445	2.256	0.9961
Surgery (y/n)	1.372	0.610	3.086	0.4442
ECOG *status*	2.278	1.102	4.706	**0.0262**
Pain score	1.450	0.696	3.022	0.3212
Visceral metastases	4.641	2.327	9.258	**< 0.0001**
Bone metastases	8.873	3.692	21.325	**< 0.0001**
*h*SUV	1.009	0.941	0.999	0.2073
Mean_10_	0.957	0.880	1.041	0.3098
TLF_10_	1.131	1.068	1.198	**< 0.0001**
	**Multivariable analysis**			
TLF_10_	1.120	1.058	1.187	**< 0.001**
PR *status*	2.266	1.015	5.061	**0.0413**

y/n = yes versus no; OS = Overall survival; HR = Hazard ratio ; CI = Confidence interval ; PR = progesterone receptor; ER = estrogen receptor.

### Relation to TTBE

Bone events occurred in 12 patients (11.2%) and these were: spinal cord compression (2 patients), pathologic bone fracture (8 patients), surgical intervention (1 patient) and intractable bone pain (1 patient). The median TTBE was 9.8 months. The presence of bone metastasis (*p* = 0.0267; HR = 4.390; 95% CI = 1.186–16.244) and negative PR *status* (*p* = 0.0227; HR = 5.406; 95% CI = 1.267–23.075) were significant risk factors for developing a bone event. No other parameters (including TLF_10_) correlated with TTBE. On multivariable analyses again only the presence of bone metastasis and negative PR *status* were significantly associated with TTBE (*p* = 0.0448 and *p* = 0.0072, respectively) (Table 4).

**Table 4:** Correlation of clinical, laboratory and imaging variables to bone event

Variables	HR	95% CI		p-value
	Univariable analyses			
Age	0.992	0.940	1.046	0.7594
Time of disease	1.097	0.989	1.217	0.0814
Primary stage (III/IV vs I/II)	1.053	0.261	4.246	0.9422
HER2	2.650	0.513	13.685	0.2445
ER status	0.996	0.121	8.200	0.9971
PR status	5.406	1.267	23.075	0.0227
Ki-67	1.009	0.969	1.051	0.6686
Radiotherapy (y/n)	-	-	-	0.0502
Hormonal therapy (y/n)	2.795	0.348	22.445	0.3336
Surgery (y/n)	-	-	-	0.5081
ECOG status	1.728	0.451	6.625	0.4252
Pain score	1.052	0.253	4.293	0.9435
Visceral metastases	1.608	0.345	7.496	0.5454
Bone metastases	4.390	1.186	16.244	0.0267
hSUV	0.995	0.967	1.024	0.7318
Mean10	0.986	0.879	1.107	0.8155
TLF10	1.082	0.987	1.186	0.0913
	**Multivariable analysis**			
Bone metastases	1.118	1.003	1.247	0.0448
PR status	10.454	1.890	57.824	0.0072

y/n = yes versus no; OS = Overall survival; HR = Hazard ratio ; CI = Confidence interval ; PR = progesterone receptor; ER = estrogen receptor.

## DISCUSSION

Previous reports have demonstrated that skeletal tumor burden on ^18^F-Fluoride PET/CT, quantified by the simple method of obtaining the TLF_10_ (SUVmax threshold = 10) is a strong and independent prognostic biomarker in prostate cancer patients undergoing ^223^Ra [9]. To our knowledge, there have not been prior studies describing that skeletal tumor burden on ^18^F-Fluoride PET/CT is an independent prognostic biomarker in breast cancer patients. Actually, the few studies conducted to identify PET parameters that predict survival in metastatic breast cancer were performed with ^18^F-FDG PET or PET/CT. These studies (with ^18^F-FDG PET/CT) have demonstrated that total lesion glycolysis bears a strong correlation to OS [13, 14]. The only other investigation evaluating the prognostic role of ^18^F-Fluoride PET/CT that we found was conducted by Piccardo *et al*. in 32 breast cancer patients [12]. Although the authors did not discover a strong and independent association of ^18^F-Fluoride PET/CT with OS, their study was the first to attempt to use semi-quantitative parameters for this purpose. The discrepancy among their findings and ours may be due to the method of tumor burden quantification. We used the TLF_10_ parameter since we have conducted extensive studies with this metrics. We established the ideal cut-off values to separate normal bone from lesions and proved it a valuable independent prognostic imaging biomarker to predict OS in prostate cancer patients [9, 15].

In the clinical setting, while it seems obvious that breast cancer patients with very low bone tumor burden will have better outcomes than those patients with high tumor burden, it is still relevant to increase awareness by a scientific approach as opposed to mere observation. We found that the median overall survival was 15.2 months for patients with bone metastasis *vs* 23.4 months for patients without bone metastasis. Visual analysis of the presence *vs* absence of bone metastases also demonstrates a significant and high likelihood of death in patients presenting with bone metastases (*p* = 0.007; HR = 6.461). However, on a multivariable model, visual analysis does not correlate with OS and only TLF_10_ can independently define which patients have worst prognosis.

We did not decide on performing ^18^F-Fluoride PET/CT over ^18^F-FDG PET/CT in these breast cancer patients. We performed ^18^F-Fluoride PET/CT over conventional bone scintigraphy because of the higher sensitivity to detect bone lesions. In fact, 18.7% of these women were also submitted to ^18^F-FDG PET/CT scans during treatment, to evaluate response to therapy. However, the determination of whole-body tumor burden ^18^F-FDG PET/CT scans using TLG and MTV parameters in metastatic breast cancer patients (especially with bone lesions) is not feasible on a daily basis. Since in breast cancer patients, osteoblastic bone metastases predominate, we envisioned that the determination of skeletal tumor burden with ^18^F-Fluoride PET/CT might be a substitute for whole-body ^18^F-FDG tumor burden calculations in daily clinical practice.

Clinical, laboratory and imaging parameters are used to prognosticate patients with limited and advanced breast cancer. However, these parameters cannot be used independently. At initial staging of patients, ECOG *status*, primary tumor histology, serum laboratory measurements, tumor markers and conventional images have relevant prognostic value. Worse prognosis is associated with absence of hormone receptors, Her2-neu gene amplification and high percentage of Ki-67 positive cells [16]. However, these variables (clinical, laboratory and imaging) could lose the ability to be independent prognostic biomarkers as the disease becomes advanced. For example, Piccardo *et al*. [12] have found that in breast cancer patients with bone metastases, the ^18^F-FDG PET/CT findings have a stronger prognostic impact in OS with an independent association than conventional clinical and biological prognostic factors. Likewise, we demonstrated that among all variables evaluated (as ECOG *status*, pain score, treatments, presence of visceral metastases, patient age, time of cancer), only the PR *status* (at initial diagnosis) and the quantitative (i.e., objective) volumetric analysis (TLF_10_) of bone tumor burden (during the course of disease) independently separated survivors from non-survivors. The mean TLF_10_ of patients that were alive at the end of follow-up was four times lower than the TLF_10_ of the 19 patients that were dead (1,562 *vs* 6,288). With a cutoff TLF_10_ value of 3,700 there was a significant difference in survival (specificity = 93.3%). Furthermore, the prognostic impact of skeletal tumor burden (TLF_10_) was high for both staging and restaging in patients with bone metastases. Therefore, since skeletal tumor burden calculation will relate to OS and PFS (in both staging and restaging settings), it may help define future therapeutic strategies.

TFL_10_ was also an independent predictor of PFS in breast cancer patients, even among patients with visceral disease progression. Using the cutoff TLF_10_ value of 1,815 discriminated patients that were more likely to progress.

Earlier studies report bone events occurring in nearly 50% of patients with breast cancer with a median TTBE of 5.5 months [17, 18]. In our population however, only 12 patients (11.2%) had bone events and among these, nine of 49 (18%) had BE due to bone metastasis; the remaining three patients (without bone metastases) developed pathological fractures because of osteoporosis during follow-up. The median TTBE in our study was 9.8 months. This discrepancy of findings between the literature and ours may be due to differences in treatment of bone metastases, nowadays with more advanced drugs that protect bones from fractures. The TLF10 value (i.e. the determination of skeletal tumor burden) was not an indicator of TTBE. However, the presence of bone metastases increased 4 times the risk of developing a bone event.

One limitation of our study was its retrospective nature with patients undergoing multiple treatment regimens. However, because of the large sample size (107 patients) we were able to evaluate the bone burden of breast cancer patients with a variety of lesions, ranging from none to a near super-scan.

## MATERIALS AND METHODS

### Study design

The local Institutional Review Board approved this retrospective study (#46/2016) of patients with breast cancer that underwent whole-body ^18^F-Fluoride PET/CT images for investigation of bone metastases.

### Patient population

Inclusion criteria consisted of histologically confirmed breast cancer patients, above 18 years, that underwent ^18^F-Fluoride PET/CT. All patients were followed-up for at least 12 months or until death. We excluded patients whose imaging study could not be retrieved and also patients lost to follow-up after the collection of the ^18^F-Fluoride PET/CT data.

### ^18^F-Fluoride PET/CT

All patients underwent a true whole-body PET/CT acquisition on two PET/CT scanners (Siemens Biograph True-Point PET/CT 64 or Siemens Biograph PET/CT 16, Siemens Healthcare, USA) 45 minutes after intravenous injection of 3.7MBq/kg of ^18^F-sodium fluoride. CT parameters included 5mm axial reconstruction and 120 kV or dose care kV tube voltage. PET images were acquired in 3-dimensional mode using 90s/bed position.

### ^18^F-Fluoride PET/CT Interpretation and Quantification

All ^18^F-Fluoride PET/CT images were blindly interpreted by three Nuclear Medicine physicians with over 12 years of experience with PET/CT images. All ^18^F-Fluoride PET/CT quantitative analyses were performed by two nuclear medicine physicians with 5 and 12 years of experience with PET/CT images, respectively.

Quantitative interpretation was performed on all ^18^F-Fluoride PET/CT images to determine whole-body skeletal tumor burden. ^18^F-Fluoride PET/CT images were quantified using METAVOL® software [19]. To calculate the skeletal tumor burden, a threshold for SUV_max_ = 10 to exclude normal bone was used, the details of the quantification is described in our previous study [15]. After processing the following parameters were automatically provided by the software:

*h*SUV: the highest SUV_max_ among all the metastases, Mean_10_: the mean SUV_max_ of all metastases, FTV_10_: the total volume of fluoride-avid bone metastases (in milliliters). This calculation is equivalent to the calculation of metabolic tumor volume (MTV) on ^18^F-FDG PET/CT images, TLF_10_: the skeletal tumor burden (VOI_10_x Mean_10_) i.e., the total activity of ^18^F-Fluoride-avid metastases. This calculation is comparable to the calculation of total lesion glycolysis (TLG) on ^18^F-FDG PET/CT images.

### Statistical analyses

The following information of each patient was correlated with the skeletal tumor burden parameters: age, years of cancer, initial clinical stage, presence of bone metastases, presence of visceral metastases, primary tumor characteristics (Ki-67, hormone receptor *status*, HER-2, histology), previous treatments and clinical evaluation using performance *status* scale (ECOG) [20] and pain scale [21]. We did not collect CA15-3 and CA27.29 values at diagnosis or to monitor recurrence because it is not recommended by the American Society of Clinical Oncology [22]. Visceral metastases were evaluated by conventional CT scans of the chest, abdomen and pelvis or by the PET/CT scans (whether with ^18^F-FDG or ^18^F-sodium fluoride).

The primary end-point was overall survival (OS), established from date of ^18^F-Fluoride PET/CT until date of death from any cause, censoring data on last follow-up of living patients. Secondary end-points were progression free-survival (PFS) and time to bone event (TTBE). PFS was defined as length of time from the ^18^F-Fluoride PET/CT image until the date of objective tumor progression or death of any cause. Objective tumor progression was defined as a new lesion (whether bone or soft tissue or visceral) or a lesion that increased in size (RECIST criteria) leading to a change in current therapy or initiation of another therapy. TTBE was defined from the date of ^18^F-Fluoride PET/CT until the date of a bone event (surgical intervention, spinal cord compression, pathologic fracture, bone pain or rapid lesion progression requiring immediate intervention).

Numerical variables were described as mean value, standard deviation, minimum and maximum and median values, and categorical variables were described with absolute and percentage frequency. To evaluate the relationship between the variables and outcomes as predictors of survival the cox proportional hazards regression was applied. ROC curve was used to determine the cutoff points for measuring the TLF_10_ and the Kaplan-Meier survival curves to demonstrate survival time distributions. The level of significance was set at 5%.

## CONCLUSIONS

The skeletal tumor burden determined with ^18^F-Fluoride-PET/CT is a powerful prognostic biomarker of OS and PFS in breast cancer patients. While the simple presence of bone metastases is associated with worst prognosis we have demonstrated that, among all patients with bone metastases, it is possible to objectively discriminate which ones will have worst outcome. This may help improve treatment strategies for breast cancer patients. To understand the relevance of our findings, more studies are necessary to evaluate if the skeletal tumor burden metrics will ultimately alter these treatment strategies.

## Abbreviations

OS: Overall survival
TLG: Total lesion glycolysis
MTV: Metabolic tumor volume
PFS: Progression free-survival
TTBE: Time to bone event
TLF_10_: Skeletal Tumor Burden
PR: Progesterone receptor

## References

[1] Glendenning J, Cook G (2013). Imaging breast cancer bone metastases: current status and future directions. Semin Nucl Med.

[2] Fogelman I, Cook G, Israel O, Van der Wall H (2005). Positron emission tomography and bone metastases. Semin Nucl Med.

[3] Yamashita K, Koyama H, Inaji H (1995). Prognostic significance of bone metastasis from breast cancer. Clin Orthop Relat Res.

[4] Coleman RE (2001). Metastatic bone disease: clinical features, pathophysiology and treatment strategies. Cancer Treat Rev.

[5] Gradishar WJ, Anderson BO, Balassanian R, Blair SL, Burstein HJ, Cyr A, Elias AD, Farrar WB, Forero A, Giordano SH, Goetz M, Goldstein LJ, Hudis CA (2015). NCCN Guidelines Insights Breast Cancer, Version 1.2016. J Natl Compr Canc Netw.

[6] Segall G, Delbeke D, Stabin MG, Even-Sapir E, Fair J, Sajdak R, Smith GT, SNM (2010). SNM practice guideline for sodium 18F-fluoride PET/CT bone scans 1.0. J Nucl Med.

[7] Bortot DC, Amorim BJ, Oki GC, Gapski SB, Santos AO, Lima MC, Etchebehere EC, Barboza MF, Mengatti J, Ramos CD (2012). 18F-Fluoride PET/CT is highly effective for excluding bone metastases even in patients with equivocal bone scintigraphy. Eur J Nucl Med Mol Imaging.

[8] Hillner BE, Siegel BA, Hanna L, Duan F, Quinn B, Shields AF (2015). 18F-fluoride PET used for treatment monitoring of systemic cancer therapy: results from the National Oncologic PET Registry. J Nucl Med.

[9] Etchebehere EC, Araujo JC, Fox PS, Swanston NM, Macapinlac HA, Rohren EM (2015). Prognostic Factors in Patients Treated with 223Ra: The Role of Skeletal Tumor Burden on Baseline 18F-Fluoride PET/CT in Predicting Overall Survival. J Nucl Med.

[10] Carkaci S, Sherman CT, Ozkan E, Adrada BE, Wei W, Rohren EM, Mawlawi OR, Ueno NT, Buchholz TA, Yang WT (2013). (18)F-FDG PET/CT predicts survival in patients with inflammatory breast cancer undergoing neoadjuvant chemotherapy. Eur J Nucl Med Mol Imaging.

[11] Gallamini A, Zwarthoed C, Borra A (2014). Positron Emission Tomography (PET) in Oncology. Cancers (Basel).

[12] Piccardo A, Puntoni M, Morbelli S, Massollo M, Bongioanni F, Paparo F, Altrinetti V, Gonella R, Gennari A, Iacozzi M, Sambuceti G, DeCensi A (2015). 18F-FDG PET/CT is a prognostic biomarker in patients affected by bone metastases from breast cancer in comparison with 18F-NaF PET/CT. Nucl Med (Stuttg).

[13] Marinelli B, Espinet-Col C, Ulaner GA, McArthur HL, Gonen M, Jochelson M, Weber WA (2016). Prognostic value of FDG PET/CT-based metabolic tumor volumes in metastatic triple negative breast cancer patients. Am J Nucl Med Mol Imaging.

[14] Son SH, Lee SW, Jeong SY, Song BI, Chae YS, Ahn BC, Lee J (2015). Whole-Body Metabolic Tumor Volume, as Determined by (18)F-FDG PET/CT, as a Prognostic Factor of Outcome for Patients With Breast Cancer Who Have Distant Metastasis. AJR Am J Roentgenol.

[15] Rohren EM, Etchebehere EC, Araujo JC, Hobbs BP, Swanston NM, Everding M, Moody T, Macapinlac HA (2015). Determination of Skeletal Tumor Burden on 18F-Fluoride PET/CT. J Nucl Med.

[16] Fitzgibbons PL, Page DL, Weaver D, Thor AD, Allred DC, Clark GM, Ruby SG, O'Malley F, Simpson JF, Connolly JL, Hayes DF, Edge SB, Lichter A, Schnitt SJ (2000). Prognostic factors in breast cancer. College of American Pathologists Consensus Statement 1999. Arch Pathol Lab Med.

[17] Jensen AØ, Jacobsen JB, Nørgaard M, Yong M, Fryzek JP, Sørensen HT (2011). Incidence of bone metastases and skeletal-related events in breast cancer patients: a population-based cohort study in Denmark. BMC Cancer.

[18] Plunkett TA, Smith P, Rubens RD (2000). Risk of complications from bone metastases in breast cancer. implications for management. Eur J Cancer.

[19] Hirata K, Kobayashi K, Wong KP, Manabe O, Surmak A, Tamaki N, Huang SC (2014). A Semi-Automated Technique Determining the Liver Standardized Uptake Value Reference for Tumor Delineation in FDG PET-CT. PLoS One.

[20] Oken MM, Creech RH, Tormey DC, Horton J, Davis TE, McFadden ET, Carbone PP (1982). Toxicity and response criteria of the Eastern Cooperative Oncology Group. Am J Clin Oncol.

[21] World Health Organization (1987). Traitement de la douleur cancéreuse. Geneva.

[22] Harris L, Fritsche H, Mennel R, Norton L, Ravdin P, Taube S, Somerfield MR, Hayes DF, Bast RC, American Society of Clinical Oncology (2007). American Society of Clinical Oncology 2007 update of recommendations for the use of tumor markers in breast cancer. J Clin Oncol.

